# The Relationship between Parental Autonomy Support and Children’s Self-Concept in China—The Role of Basic Psychological Needs

**DOI:** 10.3390/bs14050415

**Published:** 2024-05-15

**Authors:** Wei Chen, Ying Sun, Yiqing He

**Affiliations:** School of Education, Tianjin University, Tianjin 300350, China; cw1109@tju.edu.cn

**Keywords:** parental autonomy support, basic psychological needs, children’s self-concept, mental health, primary school children

## Abstract

This study aimed to investigate the relationship between parental autonomy support and children’s self-concept, and to explore the role of basic psychological needs in Chinese primary schools from the perspective of self-determination theory. A total of 3109 children aged 6–13 years participated in eastern China. The results indicated a significant correlation between parental autonomy support, basic psychological needs, and children’s self-concept. Basic psychological needs play a partial mediating role between parental autonomy support and children’s self-concept. Specifically, autonomy support varied by need types whereas parental control steadily played a negative predictive role. Parental autonomy support and control predicted children’s self-concept differently through three basic psychological needs, with differences across gender and grades. Boys and elder children had stronger relationships to competence needs, while girls were sensitive to autonomy needs; in addition, both of them were sensitive to relatedness needs. The mediating effects model and cross-group analyses revealed the complex predictive role of parental autonomy support on children’s self-concept in China, providing an effective entry point for cross-cultural research and family education to improve children’s psychological well-being.

## 1. Introduction

According to the first report on mental disorders among children and adolescents, 17.5% of Chinese children between the ages of 6 and 16 have mental health issues, and half of the problems show up early, before the age of 14 [1]. In addition, *The 2023 Blue Book on China’s Psychiatric Mental Health* points out that mental health problems in the student population are becoming increasingly prominent and are trending towards a younger age [2]. Children’s mental health issues urgently need the attention and intervention of researchers.

It has been shown that children’s self-concept reflects children’s awareness of their position in the environment and society, as well as values for evaluating themselves. It also plays a crucial role in ensuring that children develop their personality traits and reach socialization goals, which regulates psychological activities and behavior [3]. Understanding children’s self-concept can help parents or psychologists improve children’s thoughts and feelings about themselves, thereby preventing the occurrence of behavioral and emotional difficulties. Children’s self-concept begins in infancy and matures in adolescence. Negative self-concept is detrimental to children’s behavior, learning, and social functioning [4], and, in severe cases, can lead to personality distortion [5].

Family is the earliest and most far-reaching microsystem affecting children [6]. Parenting style is the external factor that has the biggest impact on an individual’s self-concept out of all the family components that affect children’s social development and mental health [7]. Deci and Ryan proposed the self-determination theory [8], which argues that the external environment facilitates the internalization of external motivation and reveals the effective ways in which external interventions can influence individual motivation. Self-determination theory assumes that everyone has an innate, intrinsic, constructive sense of self-improvement and integration, as well as a tendency to become whole with others or the surrounding society. This tendency to move from selflessness to self-development is called internalization. However, internalization does not necessarily occur but requires nourishment from the external environment to satisfy the basic psychological needs of self-development. Thus, exploring the impact of parental autonomy support on self-concept is crucial to understanding children’s development [9,10]. The present study attempts to investigate the predictive factors and formation mechanisms of contemporary children’s self-concept from the family perspective in the context of Chinese culture, to find solutions to alleviate children’s mental health problems.

### 1.1. Parental Autonomy Support and Children’s Self-Concept

Parental autonomy support is one of the important family parenting styles, which is defined as the parent’s capacity to empathize with the child’s perspective, allow children to make their choices, minimize the use of control and authoritarianism, and support the child in pursuing interests and values of their own [11]. Ryan and Deci point out that autonomy-supportive environments are conducive to individuals exploring their inner resources and developing evaluative systems, based on personality traits and interests, while internalizing external cultural values [12]. It has been found that parental autonomy support is mostly associated with positive outcomes. Parental autonomy support can make individuals feel more support and encouragement, which is not only conducive to obtaining a positive parent–child relationship [13] but also promotes the individual’s emotional adaptive functioning [14] and enhances life satisfaction [15]. Adolescents in autonomy-supportive parenting conditions are more likely to be given the freedom to handle problems on their own and receive more attention and respect, and as a result, have more positive self-evaluations [16]. According to Erikson, childhood is a critical period for the development of autonomy. Emotional or adjustment problems may occur in adulthood without the development of autonomy [17]. Parents may need to provide their children with adequate autonomy support during the parenting process, which can help foster the development of self-determined behaviors in children [18,19]. Autonomy support serves as the foundation for people to internalize and transform external values into who they are and becomes a crucial factor in encouraging people to internalize motivation [20]. Thus, we infer that autonomy support can positively predict children’s self-concept.

### 1.2. The Role of Basic Psychological Needs

Self-determination theory suggests that individuals have innate basic psychological needs, including the needs for autonomy (the perception of self-authorship of one’s actions), competence (being effective in one’s actions and attaining a sense of mastery), and relatedness (attaining a sense of belonging and relationships that are characterized by mutual caring), for self-motivation and personality integration [9]. When needs are met, they move in a positive direction towards self-improvement. Such requirements cannot be met spontaneously; instead, they must be realized with the assistance and support of the outside world [21,22,23], such as with parental support. It has been demonstrated that parental autonomy support can contribute to the positive development of individuals by satisfying basic psychological needs, which plays a mediating role [24,25]. Notably, Xu et al. found that the direct effect of parental autonomy support on adolescents’ future career planning became negative, and future education planning was no longer significant after the addition of the mediating effects of basic psychological needs and personal growth initiative to the study with 12–18-year-old middle-school students as subjects [26]. This also suggests that the influence of parental autonomy support on adolescents’ future planning does not always work well in the Chinese cultural context. These studies reveal a complex relationship between parental autonomy support and basic psychological needs in children’s development. Autonomy-supportive parenting behaviors exercise an influence on a child’s development by providing them with the necessary conditions to satisfy psychological needs for autonomy, competence, and relatedness, whereas controlling parenting styles are often perceived as hindering children’s basic needs and leading to problematic behaviors [27]. Nevertheless, it is not clear whether basic needs satisfaction promotes the development of children’s self-concept, and whether autonomy, competence, and relatedness needs play different roles between parental autonomy support and children’s self-concept.

### 1.3. The Context of Culture

Gender and grade differences are two of the focuses of this study. When sorting out the existing studies, we find that there exist inconsistencies between conclusions with cross-group and cross-cultural variability [28,29]. Even at the same age in the same culture, there are different conclusions [5]. Freeman’s findings support the idea that the developmental curve of self-concept is undulating, declining from elementary to middle school, year to year [30]. Marsh’s research with children aged 6–18 years found that children’s self-concept follows a U-shaped curve [31]. Su et al. found that 7–9-year-olds scored lower than 10–16-year-olds on some dimensions of self-concept, such as behavior, popularity, happiness, and satisfaction, and higher than elder children on general and academic status and anxiety [32]. A study with a sample of Greek children showed no significant differences in self-concept by gender, nor was there an interaction between gender and grade level.

Additionally, research on autonomy support is culturally controversial. Self-determination theory views issues of autonomy as key to understanding human behavior and as a fundamental human need. Cultures may differ in their emphasis on autonomy and independence, but the need for autonomy is universal, and the fact that optimal well-being is experienced through the satisfaction of needs is independent of culture [21,33]. In contrast, scholars with a culturally particular perspective contend that the negative impact of parents not providing autonomy to children may be less in the context of East Asian cultures than in Western cultures. East Asian parents are more likely to be accepted by each other for making decisions for their children in societies that value collectivism and interdependence [34]. This is especially true in Chinese culture, which is greatly inspired by Confucianism, which emphasizes the importance of relationships. A society that places a high priority on human connectedness and interdependence is less likely to violate children’s sense of self through parental control [35]. The traditional cultural atmosphere in Chinese society of “hoping that their children will become loong and daughters become phoenixes” has led parents regarding the upbringing of their offspring as a responsibility and an obligation that they should fulfill, to give priority to their children in the allocation of resources, and to do everything in their power to support them. Chao found that Chinese children are less likely to rebel against parental discipline and perceive it as their parents’ care and concern for them [36].

### 1.4. Present Study

**Figure 1:**
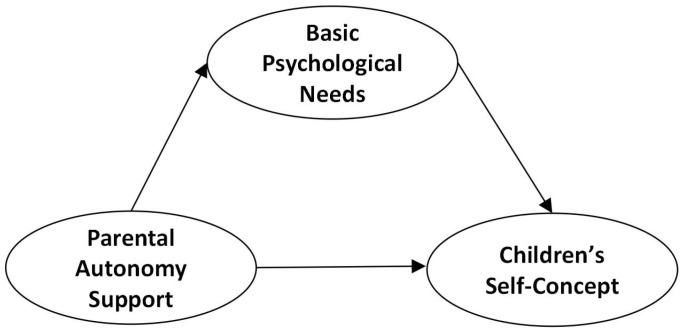
Research hypothesis model M_1_.

Empirical studies on the prediction of children’s physical and mental development by parental autonomy support have mostly focused on adolescents and college students in China, with fewer studies on children, which mainly focus on the upper grades of primary school and pay less attention to children in the lower grades. No researcher has yet explored the role of parental autonomy support and basic psychological needs satisfaction in the formation of children’s self-concept within the self-determination theory. The present study attempts to overcome the limitations of earlier empirical research that did not include children in younger primary grades. Preliminarily, we explored the relationship between parental autonomy support, basic psychological needs, and children’s self-concept, as well as their gender and grade differences in the Chinese cultural context, and verified the role of basic psychological needs. The theoretical hypothetical model M_1_ (Figure 1) is as follows.

## 2. Materials and Methods

### 2.1. Participants and Procedure

Children were recruited as subjects from primary schools in Shandong Province, East China. Recruitment began in October 2023 and ended in December. A total of 3350 children participated in this study. After excluding questionnaires that were garbled or omitted, 3109 questionnaires were obtained, with a validity rate of 92.8%. Of these, 52.8% were boys and 47.2% were girls; 33.7% were in grade one, 20.9% were in grade two, 18.2% were in grade three, 13.4% were in grade four, and 13.8% were in grade five, with ages ranging from 6 to 13 years (*M* = 8.3, *SD* = 1.58).

Before administering the questionnaire, the experimenter received uniform instructions to administer the test as a class. Children gave informed consent to complete the test voluntarily and could withdraw from the survey at any time. This study was approved by the local school authorities and schools, and informed consent was obtained from the students and their parents. Further, the study was approved by the Ethics Committee of Tianjin University (TJUE-2021-173) and complied with the standards of the Declaration of Helsinki on research ethics. The average duration of the test was approximately 30 min with the length of the test depending on the age and cognitive level of children. For children in the lower primary grades (e.g., grades 1 and 2) who were less literate and had poorer comprehension skills, trained administrators such as classroom teachers and graduate students in psychology, read the questions aloud to them individually to ensure that they were able to answer them completely. Data were processed and analyzed using the SPSS 27.0 and AMOS 24.0 statistical software.

### 2.2. Measures

#### 2.2.1. Parental Autonomy Support

The Parental Autonomy Support Questionnaire was developed by Wang et al. [34], and the Chinese version was revised by Zu [37]. There were 24 items and two dimensions: autonomy support and parental control. Autonomy support was examined in terms of choice-making and opinion exchange, which was positively scored, and parental control was categorized as guilt induction, love withdrawal, and authority assertion, which was negatively scored. A 5-point Likert scale was adopted. Higher total scores represent higher levels of parental support and parental control. The Cronbach’s alpha coefficient in each dimension ranged from 0.91 to 0.93. Confirmatory factor analysis results revealed a good model fit: *χ*^2^/*df* = 11.572, RMSEA = 0.058, GFI = 0.922, AGFI = 0.904, CFI = 0.938, TLI = 0.930, IFI = 0.938, and standardized factor loadings ranged from 0.50 to 0.81. The CR coefficients ranged from 0.92 to 0.93, and the AVE coefficients ranged from 0.47 to 0.58. The results indicate that the measurement model has overall good reliability and validity.

#### 2.2.2. Basic Psychological Needs

The Basic Psychological Needs Scale was developed by Gagné [38] and the Chinese version was revised by Liu et al. [39]. There were 19 items and three dimensions: the needs for autonomy, competence, and relatedness. There were 6–7 items per subscale, 9 items of which are reverse scored. A 7-point Likert scale was adopted. Higher scores represent higher levels of psychological needs satisfaction in children. The Cronbach’s alpha coefficient in each dimension ranged from 0.62 to 0.74. Confirmatory factor analysis results revealed a good model fit: *χ*^2^/*df* = 11.235, RMSEA = 0.057, GFI = 0.957, AGFI = 0.933, CFI = 0.935, TLI = 0.910, IFI = 0.936, and standardized factor loadings ranged from 0.59 to 0.73. The CR coefficients ranged from 0.67 to 0.72, and the AVE coefficients ranged from 0.41 to 0.46. The results indicate that the measurement model has overall acceptable reliability and validity.

#### 2.2.3. Children’s Self-Concept

The Children’s Self-Concept Scale was developed by Piers and Harris, and the Chinese version was revised by Su et al. [5]. The scale consists of 80 items and contains six subscales: behavior, general and academic status, physical appearance and attributes, anxiety, popularity, and happiness and satisfaction. Each subscale has 10–17 items, nearly half of which are reverse-scored. The scale is positively scored according to yes and no responses, with higher scores representing a higher level of self-concept in children. The Cronbach’s alpha coefficient was 0.887. Confirmatory factor analysis results revealed a good model fit: *χ*^2^/*df* = 10.953, RMSEA = 0.057, GFI = 0.994, AGFI = 0.976, CFI = 0.994, TLI = 0.982, IFI = 0.994, and standardized factor loadings ranged from 0.50 to 0.76. The CR coefficient was 0.89, and the AVE coefficient was 0.57. The results indicate that the measurement model has overall good reliability and validity.

## 3. Results

### 3.1. Common Method Biases Test

Self-reported data were used in this study. There may be an issue of common methodological bias; despite having been controlled during the administration of tests by using forward and reverse scoring, further statistical tests need to be carried out. Harman’s single-factor test found that the first principal component factor approach explains 14.775%, which is less than the criterion of 40% [40]. A confirmatory factor analysis was then used to test for common method biases for all self-reported items [41], which showed that the one-factor model fitted the indicators poorly: *χ*^2^/*df* = 163.466, RMSEA = 0.229, GFI = 0.596, AGFI = 0.416, CFI = 0.643, NFI = 0.642, TLI = 0.564, and IFI = 0.643. Consequently, no obvious issues of common methodological bias were found in this research.

### 3.2. Descriptive Statistics and Correlation Analysis

Pearson’s correlation was used to analyze the correlation between parental autonomy support, basic psychological needs, and children’s self-concept. Table 1 shows that parental autonomy support and basic psychological needs are significantly and positively correlated with children’s self-concept, and parental control was significantly and negatively related to them.

### 3.3. Children’s Self-Concept Measurement Invariance across Gender and Grade

Considering self-concept may be undergoing developmental changes between gender groups, as well as between grades, we verified configural (factor structure) and metric (factor loading) invariance for the children’s self-concept scale first. The comparison across gender and grade (first and second grades for the younger age group and third to fifth grades for the elder age group) of measurement invariance indicated that the models were well-fitted(see Table S1). The change in CFI and RMSEA did not exceed the recommended thresholds (ΔCFI ≤ 0.01, ΔRMSEA ≤ 0.01) [42], indicating that the self-concept scales satisfy cross-sample invariance.

**Figure 2:**
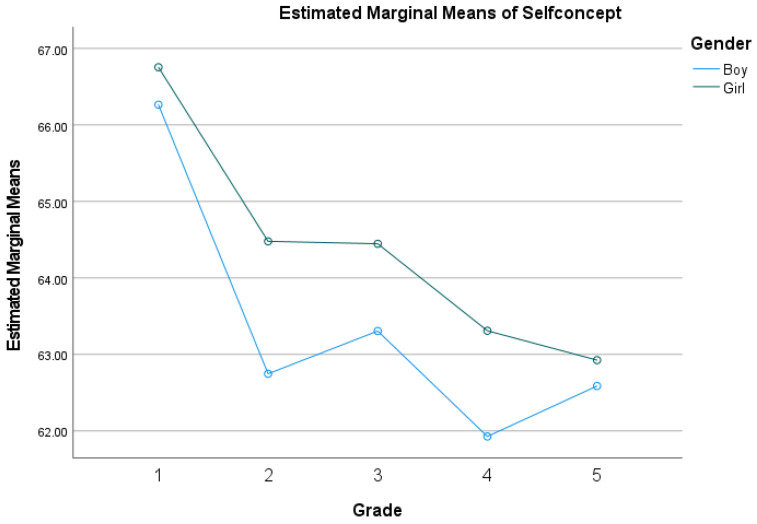
The distribution of children’s self-concept by gender and grade.

Based on measurement invariance, we performed independent sample *t*-tests (Table 2). Notably, we did not find gender differences in parental autonomy support and relatedness needs, nor did find grade differences in parental control. Boys felt more parental control compared to girls and scored lower on most variables, and younger children scored higher than elder children. Figure 2, which displays the distribution of children’s self-concept by gender and grade, also supports this result. A two-way ANOVA was conducted with self-concept as the dependent variable and gender and grade as the independent variables. The results showed significant main effects of gender (*F* = 14.11, *p* < 0.001) and grade (*F* = 61.91, *p* < 0.001), with no interaction between them (*F* = 0.122, *p* > 0.05). In terms of main effects, boys were significantly lower on self-concept than girls, with children in the younger age group having significantly higher levels than those in the elder age group. Thus, it was necessary to regard them as cross-group variables in subsequent analyses.

### 3.4. General Mediating Effects Analysis of Basic Psychological Needs

**Figure 3:**
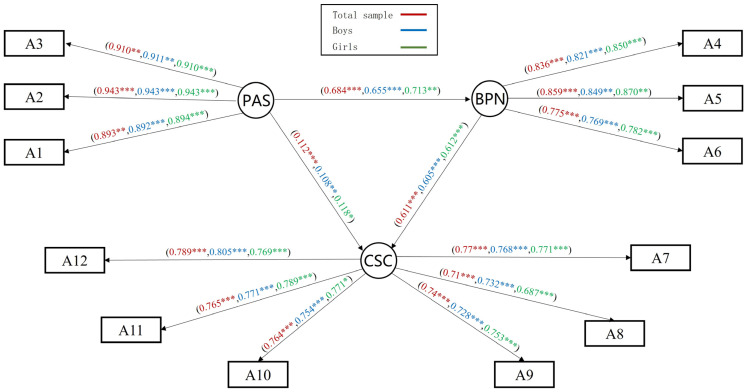
General mediation effects model of basic psychological needs M_G_. Note. Red represents the standardized path coefficients for the total sample, blue for the boys’ group, and green for the girls’ group. * *p* < 0.05, ** *p* < 0.01, *** *p* < 0.001.

We tested the standardized path coefficients using a maximum likelihood method to explore the general mediating effect of parental autonomy support on children’s self-concept. It was found that all paths were significant. The M_G_ fit indices were good: *χ*^2^/*df* = 10.249, RMSEA = 0.055, NFI = 0.979, IFI = 0.981, GFI = 0.972, AGFI = 0.957, CFI = 0.981, and TLI = 0.976. Parental autonomy support can predict children’s self-concept by basic psychological needs (Figure 3). Subsequently, we conducted a mediation effect test using a bias-corrected bootstrap method (sample size of 5000) [43]. The results showed that the confidence interval for the mediating effect did not contain 0, the mediation model was significant, and the effect size of the indirect effect was 78.8% of the total effect. The 95% confidence intervals and effect values for the mediation model are shown in Table 3.

Multi-group analysis was used to explore whether there was structural consistency across gender in the general mediating model. Unconstrained, measurement weights, and structural weights models were constructed to compare differences, which showed that all the fitting indexes of each model were acceptable (see Table S2) [44]. Further model invariance tests showed that the change in model fit indicators reached significance compared to the unconstrained model (*p* < 0.05), with significant cross-group differences between boys and girls (see Table S2). Since the above illustration is a holistic undifferentiated *χ^2^* test that may obscure the between-group effects of specific factor loadings, the differences in the critical ratio values (CR) of path coefficient comparisons between groups are also examined [45]. There was a significant gender difference in the coefficient value for parental autonomy support for basic psychological needs (CR = 2.502, *p* < 0.01). The results of the bias-corrected bootstrap-mediated effects test indicate that both of the mediated models were significant (Table 3). Furthermore, multi-group analysis of grade showed that the between-group differences in mediation models and differences in the CR comparison of path coefficients were not significant (*p* > 0.05). Therefore, we do not discuss it here.

The results of the general mediation model support the predictive role of parental autonomy support and basic psychological needs on children’s self-concept: that boys and girls differ in perceived parental autonomy support and satisfaction of basic psychological needs. However, analyses of single latent variables may obscure specific internal information. Thus, we conducted separate analyses of the mediating effects.

### 3.5. Mediating Effects Analysis and Multi-Group Test

Based on the above studies, we used structural equation modeling for parental support and parental control, three basic psychological needs (autonomy/competence/relatedness needs) for separate mediation model estimation, and cross-group analyses to check the standardized path coefficients. Tests of mediating effects were conducted using the bias-corrected bootstrap method. The 95% confidence intervals and effect values are shown in Table 4. Model comparison fitness indexes and model invariance results are shown in Table S3. We found that different basic psychological needs predicted children’s self-concept differently.

#### 3.5.1. Analysis of the Mediating Effect of Autonomy Needs and Multi-Group Test

**Figure 4:**
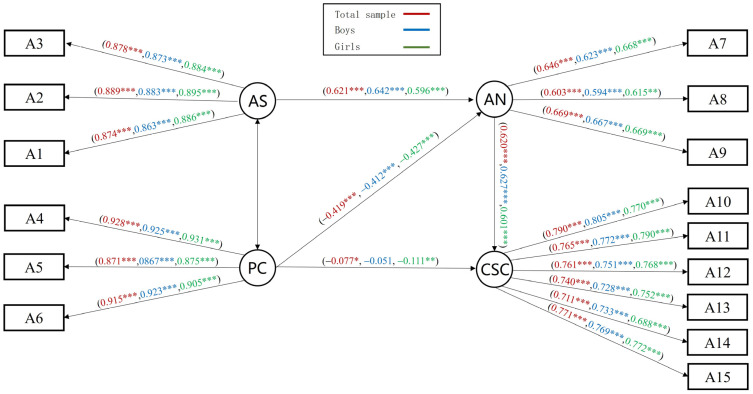
Mediating effect model of autonomy needs, M_AN_. Note. * *p* < 0.05, ** *p* < 0.01, *** *p* < 0.001.

Results for the mediation model found that the path from parental support to self-concept was nonsignificant, and after deletion and refitting, the M_AN_ model fitted well: *χ*^2^/*df* = 9.445, RMSEA = 0.052, NFI = 0.972, IFI = 0.975, GFI = 0.967, AGFI = 0.953, CFI = 0.975, and TLI = 0.969. Further tests of mediating effects found that autonomy needs fully mediated parental support and self-concept and partially mediated parental control and self-concept. Parental support positively predicted self-concept through needs for autonomy, while parental control played an inhibitory role (Figure 4).

A comparison across groups for gender and grades was conducted next. The findings of the cross-group analyses for grades indicated that the between-group differences in the mediation model comparison and the differences in the CR values of path coefficients were not significant (*p* > 0.05). The results of gender indicated gender differences in the predictive effect of parental control on self-concept, and in the boys’ group, the direct predictive effect of parental control on self-concept was not significant. Specific examination of the difference in CR values between the two groups [45] revealed that there was a significant difference in the path from autonomy needs to self-concept (CR = −2.729, *p* < 0.01). The specific standardized path coefficients are shown in Model M_AN_.

#### 3.5.2. Analysis of the Mediating Effect of Competence Needs and Multi-Group Test

**Figure 5:**
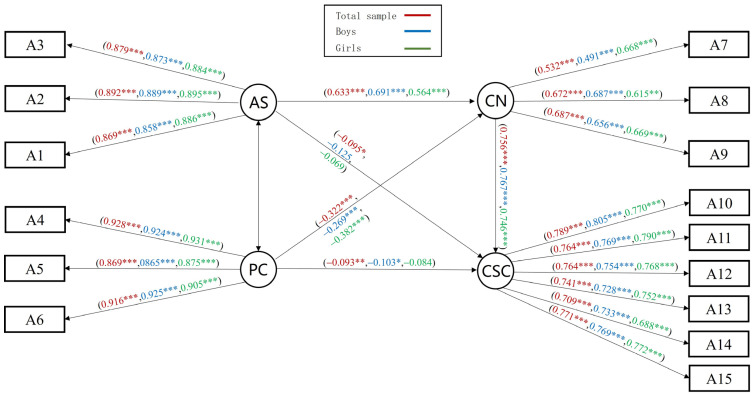
Mediating effect model of competence needs, M_CN(gender)_. Note. * *p* < 0.05, ** *p* < 0.01, *** *p* < 0.001.

Results of the mediation model of needs for competence showed all paths to be significant. Both parental support and parental control negatively predicted self-concept. The M_CN_ model fitted well: *χ*^2^/*df* = 18.475, RMSEA = 0.075, NFI = 0.947, IFI = 0.949, GFI = 0.934, AGFI = 0.906, CFI = 0.949 and TLI = 0.937. Further mediation effect tests indicated that the need for competence partially mediated parental support/parental control and self-concept (Figure 5).

**Figure 6:**
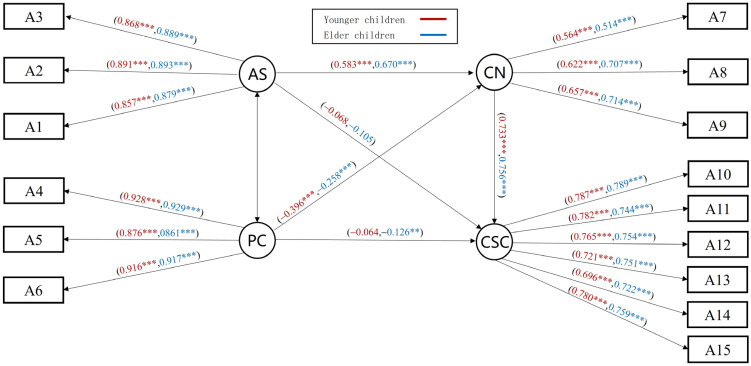
Mediating effect model of competence needs, M_CN(grade)_. Note. ** *p* < 0.01, *** *p* < 0.001.

A comparison across groups for gender and grades was conducted. Results indicated that for the boys’ group, the direct predictive effect of parental support on self-concept was not significant; with the girls’ group, both parental support and parental control on self-concept were not significant. Further comparisons of CR values showed significant gender differences in the path from parental control to competence needs (CR = −4.131, *p* < 0.001), and competence needs to self-concept (CR = −2.251, *p* < 0.01). The specific standardized path coefficients are shown in Model M_CN(gender)_ (Figure 5). Similarly, multi-group tests across grades showed that although comparisons between models showed no between-group differences (*p* > 0.5), further comparisons of CR revealed a significant difference in parental control to competence needs (CR = 4.067, *p* < 0.01), which was higher in younger children than in elder children. In addition, parental support and parental control on self-concept were not significant in the younger children group, while in the elder children group, parental support on self-concept was not significant and control was significant (Figure 6). The specific standardized path coefficients are shown in Model M_CN(grade)_.

#### 3.5.3. Analysis of the Mediating Effect of Relatedness Needs and Multi-Group Test

**Figure 7:**
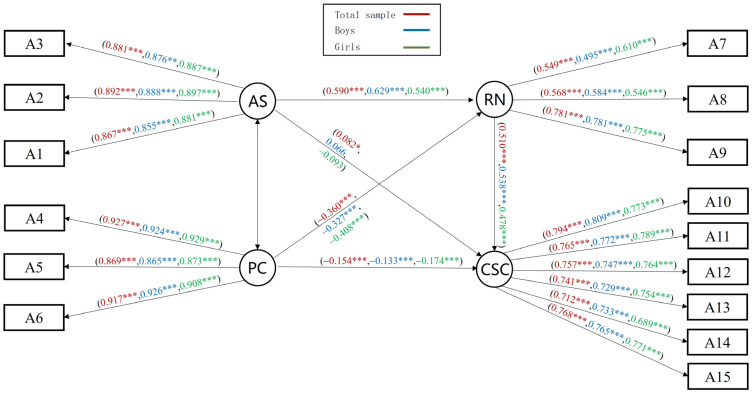
The mediating effect model of relatedness needs, M_RN_. Note. * *p* < 0.05, ** *p* < 0.01, *** *p* < 0.001.

The results of the mediation model for the relatedness needs showed a positive and significant path from parental support to self-concept, with parental control being a negative predictor. The M_RN_ fitted well: *χ*^2^/*df* = 15.771, RMSEA = 0.069, NFI = 0.954, IFI = 0.957, GFI = 0.945, AGFI = 0.921, CFI = 0.957, and TLI = 0.946. Further mediation effect tests showed significant mediation effects, with the need for relatedness partially mediating parental support/parental control and self-concept (Figure 7).

A comparison across groups for gender and grades was conducted. Findings from the analyses for grade indicated that neither between-group model differences nor the differences in the CR values for the model path coefficients were significant (*p* > 0.05), which means no grade difference. The results of the gender cross-group analysis indicated that the direct predictive effect of parental support on self-concept was not significant in both boys’ and girls’ groups. Comparisons of CR values showed significant gender differences in parental control to relatedness needs (CR = −4.087, *p* < 0.001), and relatedness needs to self-concept (CR = −2.827, *p* < 0.01), as shown in the standardized path coefficients in Model M_RN_ (Figure 7).

## 4. Discussion

### 4.1. Developmental Features of Children’s Self-Concept in Chinese Primary Schools

We found significant positive associations between parental autonomy support, basic psychological needs, and children’s self-concept in this research. Similar to existing findings, parental autonomy support has often been found to be associated with positive outcomes, such as autonomy and well-being [46,47], and facilitates children’s internalization of rules and guidance [48]. This result is consistent with developmental contextualism and supports the self-determination theory [49], which states that environmental family factors play a crucial role, and that parental autonomy support is one of the most important external factors in the positive development of children [50]. Basic psychological needs are well satisfied when children feel supported and accepted in the family. As parents give autonomy to children, they feel more approval and support, which contributes to good parent–child relationships, as well as increased life satisfaction, and positive emotional experiences and self-evaluations [13,51]. Unlike previous studies [30,31], we found the developmental curve of self-concept is biased W-shaped. In particular, there was a significant turning point in the level of self-concept among children in the third grade. The trends did not follow a straight line but fluctuated in a roundabout way, and there was no interaction between gender and grades. Contrary to the findings of Piers [52] and Su [5], we found not only gender differences but also developmental differences in grades among Chinese children, with boys’ self-concept being significantly lower than girls’, and younger children being significantly lower than elder children in primary school. Boys were more likely to feel parental control, whereas there was no significant difference in parental support.

A possible reason is that the content and structure of self-concept changes with each stage of self-development, which is both stable and malleable in different environments [53]. Children started primary school with positive thoughts and feelings about themselves, but later in childhood, self-evaluation began to fluctuate. China has been implementing the “Double Reduction” policy (reducing the burden of homework and out-of-school training for students in compulsory education) in recent years, which aims to reduce the pressure on children’s schoolwork. Children in the lower grades are less stressed, grow up in a relatively tolerant environment, and are prone to evaluate themselves optimistically with positive experiences. Later, as children’s self-concept develops gradually into pre-adolescence, with cognitive enhancement children, are able to understand the multiple causes of their behavior. Coupled with the challenges of schoolwork, self-regulation and adaptability subsequently increase, leading to a decline and leveling off of self-concept, which in turn becomes progressively more objective. Moreover, Chinese Confucianism advocates that “loving mothers are more likely to fail their children”. Parents change their parenting style from simple spoiling to a more rational and complex upbringing style that gradually incorporates rules and regulations as children grow up, which is relatively stricter for boys. Meanwhile, the social and cultural gender role of boys as strong and courageous may result in boys being subjected to harsher parental discipline than girls, leading to lower self-concept, which also suggests that we should pay more attention to the situation of boys, particularly.

### 4.2. Mechanistic Analysis of Children’s Self-Concept: Role of Basic Psychological Needs

Progressive results from structural equation models indicated that basic psychological needs mediated the relationship between parental autonomy support and children’s self-concept. Different types of needs showed variations in the predictive roles.

The present study found that parental autonomy support can contribute to the development of self-concept by satisfying children’s basic psychological needs, which supports the self-determinism theory [54]. Self-determination theory suggests that every person has needs related to competence, autonomy, and relatedness. When these needs are met, individuals progress positively, and the environment can either support or hinder the individual’s innate tendency towards positive commitment and psychological growth [55]. Parental autonomy support, as an environmental factor, can only be manifested by acting on intrinsic factors within children. In self-determination theory, autonomy support works by satisfying basic psychological needs. Numerous empirical studies have also confirmed that individuals’ basic needs can be met in a good family environment [56]. The degree to which an individual’s basic needs are met can also be positively correlated with the harmony of families and the quality of family systems [57]. Moreover, satisfaction with basic psychological needs can also promote positive development, enabling them to experience positive emotions, and increase their evaluation of themselves and life satisfaction [58]. Thus, basic psychological needs are often shown to be a mediating variable between autonomy support and positive psychological traits [59]. Beyond this, parental autonomy support (autonomy support and parental control) and different types of basic psychological needs (autonomy/competence/relatedness) predicted children’s self-concepts through different channels, showing multiple complexities and cross-group variability.

First, the direct predictive role of parental support (autonomy support and parental control) on children’s self-concept varied across needs, and predictions are not in the same direction. Autonomy support was a non-significant predictor of autonomy needs, a negative predictor of competence needs, and a positive predictor of relatedness needs, with parental control consistently a negative predictor. Although it has been argued that the absence of autonomous parenting in collectivist cultures, which involves not only parental strictness and control, but also parental love and involvement, does not necessarily lead to negative consequences [36,60], and autonomy support is not important and can even be detrimental to a person’s well-being [61], we found that parental control presented no differences in children’s self-concept depending on three basic psychological needs, but instead showed a stable negative predictive effect. Despite the native parenting perspective of love and blame that embraces both caring and authority, parental control is still perceived by children as a negative parenting style that restricts their self-development. It seems that even though the supportive behaviors and psychological needs provided by parents buffer children’s self-concept development, they are not enough to counteract the destructive effects of parental control. The results of this study, to some extent, support the cultural universal view [21] that interpretations of autonomy may be different but the autonomy need is universal in both collectivist and individualist cultures, and psychological control, which can bring stressful experiences and intrusiveness to children, harms children’s development. Moreover, we found that autonomy-supportive parenting may have a poor association with children’s competence needs and self-concept. A possible explanation is that parents may lack effective monitoring while giving autonomy support, and excessive support undermines children’s self-efficacy. Consequently, it is suggested that parents should not only give autonomy support but also be appropriately involved and provide rules in their parenting, which may be the ideal way to promote the development of children’s self-concept.

Second, we seem to see that boys and girls feel differently towards the three basic psychological needs through cross-group analyses. Girls are more sensitive to the satisfaction of autonomy needs compared to boys in the development of self-concept, while boys focus on competence needs; both are sensitive to relatedness needs. The above results reveal the necessity to generalize the effects of parenting styles with an integrated perspective. Developing autonomy is a fundamental task of middle childhood and adolescence, and part of the developmental process involves parents exercising authority and parenting styles with children [62]. On the one hand, autonomy support and parental control form mutually constraining forces that are linked to children’s self-concept by jointly predicting the satisfaction of basic psychological needs. Consistent with the stress-buffering model, autonomy-supportive parenting can alleviate the negative effects of psychological control [63], in which parenting styles as a whole collectively influence the performance and development of children’s behavior. On the other hand, according to social role theory, parents have different role expectations for boys and girls, usually higher achievement expectations for boys, and higher family role expectations for girls, which also leads them to treat children differently [64]. For example, it has been suggested that girls may have greater decision-making autonomy than boys, which has been related to relative maturity [65] and may be attributed to the fact that girls tend to exhibit less problematic behavior and better academic performance [66], with parents being correspondingly more supportive and less controlling than with boys. Highly controlling parents often make decisions for children and demand compliance, which reduces children’s opportunities to solve problems independently or cope with challenging situations, reducing their ability to develop a sense of competence and efficacy [67]. In addition, we found that both boys and girls showed a desire for relatedness needs. Children who have a close relationship with their parents usually feel more secure and confident [68]. Although strong autonomy arises with the awakening of self-awareness, it is crucial for parental support, as a significant other in the development of children’s self-concept, to provide a stable and reliable safe space to satisfy children’s relational needs and motivate them to positively complete their self-integration to express positive self-appraisal.

Third, we found that parental autonomy support and control of younger children was predictive of self-concept only through the need for competence, whereas parental control was also directly negatively predictive in elder children. It is well known that children in the upper primary grades are in the pre-adolescent stage, and they are increasingly eager to be independent and to be able to obtain sufficient understanding and support from their parents. According to developmental psychology, self-concept at this stage is characterized by social appearance, which means that children value competence and achievement and judge themselves based on them [69,70]. However, due to the lack of cognitive ability and social experience, as well as the pressure to enter the next school year, parents inevitably impose restrictions and discipline on their lives and studies. Children with a certain sense of defiance and independence during this period may perceive parental life and emotional care as more parental psychological control and see them as obstacles to gaining a sense of self-efficacy, leading to frustration with basic psychological needs for competence. Parents facing groups of elderly children may have to explore different parenting styles, focusing not only on creating an autonomy-supportive family atmosphere but also on the children’s perceived feelings of autonomy support from parents, as well as focusing on the satisfaction of basic needs, especially competence needs, to help them develop their self-concept and complete developmental tasks successfully.

Therefore, the match between family parenting styles (autonomy support and parental control) and children’s basic psychological needs for growing autonomy, competence, and relatedness is crucial for the development of self-concept. Children’s desires for basic psychological needs vary across gender and grade levels. If these basic needs are satisfied by the family environment in which the individual lives, the child will develop in a positive direction. This suggests that in clinical interventions, we need to dialectically view the role of parental autonomy support in children’s self-concept and explore the positive elements involved.

## 5. Limitations

This study explored the relationship between parental autonomy support, basic needs satisfaction, and self-concept in a cross-sectional study with a sample of Chinese primary school children. Follow-up studies may further explore the effects of parental autonomy support on children’s self-concept in clinical samples through longitudinal studies to reveal complex developmental mechanisms. Secondly, we examined the role of parental autonomy support but did not differentiate between the gender of the parents to specifically explore the differences between paternal autonomy support and maternal autonomy support, which can be explored in more detail in future studies. In addition, this investigation confirmed that the positive effects of parental autonomy support on child development also apply to the Chinese cultural context, supporting the argument that the need for autonomy is universal. More in-depth difference studies can be continued in cross-cultural samples to reveal the role that cultural context plays in this regard in the future.

## 6. Conclusions

Based on self-determination theory, the present study explored the relationship between parental autonomy support, basic psychological needs, and children’s self-concept in the context of Chinese culture with primary school children, comparing gender and grade differences, and found the following:

(1) The general mediation model was significant, with basic psychological needs playing a partial mediating role between total parental autonomy support and children’s self-concept.
(2) Parental autonomy support and control predicted children’s self-concept differently across three mediating models of basic psychological needs: autonomy support varied by need types whereas parental control steadily played a negative predictive role.
(3) Different mediation models of basic psychological needs showed differences across gender and grade groups: boys and elder children were more sensitive to competence needs, with girls bring more sensitive to autonomy needs, and both of them were being sensitive to the need for relatedness.
(4) Parental autonomy support was validated as a predictor of children’s self-concept in Chinese culture.

## Figures and Tables

**Table 1:** Descriptive statistics and correlations of all variables (*n* = 3109).

Variable	1	2	3	4	5	6	7	8	9	10	11	12	13	14	M ± SD
1 PAS	1														91.36 ± 16.93
2 AS	0.534 **	1													33.82 ± 5.88
3 PC	−0.941 **	−0.215 **	1												38.45 ± 14.66
4 BPN	0.616 **	0.613 **	−0.465 **	1											16.73 ± 2.53
5 AN	0.584 **	0.558 **	−0.450 **	0.892 **	1										5.22 ± 0.98
6 CN	0.540 **	0.518 **	−0.416 **	0.903 **	0.720 **	1									5.52 ± 0.98
7 RN	0.510 **	0.553 **	−0.367 **	0.861 **	0.638 **	0.671 **	1								5.99 ± 0.90
8 CSC	0.483 **	0.411 **	−0.392 **	0.604 **	0.521 **	0.574 **	0.509 **	1							64.35 ± 8.86
9 BEH	0.463 **	0.300 **	−0.414 **	0.454 **	0.398 **	0.428 **	0.380 **	0.769 **	1						14.34 ± 2.01
10 GAS	0.379 **	0.373 **	−0.288 **	0.530 **	0.451 **	0.531 **	0.424 **	0.837 **	0.550 **	1					12.92 ± 2.92
11 PAA	0.275 **	0.343 **	−0.180 **	0.408 **	0.333 **	0.394 **	0.358 **	0.676 **	0.336 **	0.662 **	1				10.51 ± 2.50
12 ANX	0.368 **	0.272 **	−0.315 **	0.459 **	0.427 **	0.435 **	0.353 **	0.740 **	0.541 **	0.513 **	0.234 **	1			9.87 ± 2.63
13 POP	0.345 **	0.257 **	−0.296 **	0.456 **	0.398 **	0.413 **	0.402 **	0.752 **	0.533 **	0.552 **	0.381 **	0.675 **	1		9.42 ± 1.69
14 HPS	0.309 **	0.290 **	−0.241 **	0.365 **	0.297 **	0.335 **	0.339 **	0.621 **	0.442 **	0.447 **	0.574 **	0.365 **	0.383 **	1	8.67 ± 1.11

Note. Parents’ autonomy support (PAS), autonomy support (AS), parental control (PC), basic psychological needs (BPN), autonomy needs (AN), competence needs (CN), relatedness needs (RN), children’s self-concept (CSC), behavior (BEH), general and academic status (GAS), physical appearance and attributes (PAA), anxiety (ANX), popularity (POP), happiness and satisfaction (HPS). ** *p* < 0.01.

**Table 2:** Difference comparisons across gender and grade (*n* = 3109).

Variable		Gender		Grade		Variable		Gender		Grade	
		Boy (*n* = 1642)	Girl (*n* = 1467)	Younger (*n* = 1696)	Elder (*n* = 1413)			Boy (*n* = 1642)	Girl (*n* = 1467)	Younger (*n* = 1696)	Elder (*n* = 1413)
PAS	M ± SD	90.36 ± 16.83	92.49 ± 16.98	92.14 ± 16.62	90.43 ± 17.26	AN	M ± SD	5.17 ± 0.96	5.28 ± 0.99	5.27 ± 0.97	5.17 ± 0.99
	*t*/*p*	*t* = −3.50 *p* < 0.001		*t* = 2.82 *p* < 0.01			*t*/*p*	*t* = −3.13 *p* < 0.01		*t* = 2.89 *p* < 0.01	
	Cohen *d*	−0.126		0.10			Cohen *d*	−0.11		0.10	
AS	M ± SD	33.64 ± 5.90	34.01 ± 5.85	34.21 ± 5.65	33.34 ± 6.12	CN	M ± SD	5.49 ± 0.97	5.57 ± 0.98	5.61 ± 0.94	5.42 ± 1.01
	*t*/*p*	*p* > 0.05		*t* = 4.08 *p* < 0.001			*t*/*p*	*t* = −2.25 *p* < 0.05		*t* = 5.38 *p* < 0.001	
	Cohen *d*	-		0.15			Cohen *d*	−0.08		0.20	
PC	M ± SD	39.28 ± 14.77	37.53 ± 14.49	38.07 ± 14.63	38.92 ± 14.70	RN	M ± SD	5.96 ± 0.89	6.02 ± 0.90	6.07 ± 0.86	5.89 ± 0.93
	*t*/*p*	*t* = 3.34 *p* < 0.001		*p* > 0.05			*t*/*p*	*p* > 0.05		t = 5.51 *p* < 0.001	
	Cohen *d*	−0.12		-			Cohen *d*	-		0.20	
BPN	M ± SD	16.62 ± 2.49	16.86 ± 2.57	16.95 ± 2.44	16.48 ± 2.61	CSC	M ± SD	63.83 ± 9.01	64.93 ± 8.67	65.35 ± 8.54	63.15 ± 9.10
	*t*/*p*	*t* = −2.71 *p* < 0.01		*t* = 5.18 *p* < 0.001			*t*/*p*	*t* = −3.44 *p* < 0.001		*t* = 6.92 *p* < 0.001	
	Cohen *d*	−0.10		0.19			Cohen *d*	−0.12		0.25	

**Table 3:** The 95% confidence intervals and effect values for the mediation model.

Path	Total Sample			Boys			Girls		
	Effect Value	95%CI	*P*	Effect Value	95%CI	*P*	Effect Value	95%CI	*P*
PAS→BPN→CSC	0.360	[0.322,0.403]	0.000	0.358	[0.303,0.421]	0.000	0.357	[0.305,0.417]	0.000
PAS→CSC	0.112	[0.060,0.162]	0.000	0.108	[0.043,0.175]	0.001	0.118	[0.039,0.193]	0.000
Total effect	0.457	[0.420,0.497]	0.000	0.454	[0.380,0.536]	0.000	0.454	[0.405,0.506]	0.000

**Table 4:** The 95% confidence intervals for the mediation effect and the effect values.

Path		Total Sample			Boys			Girls		
		Effect Value	95% CI	*P*	Effect Value	95% CI	*P*	Effect Value	95% CI	*P*
AS→AN→CSC	M_AN_	0.347	[0.312,0.387]	0.000	0.378	[0.327,0.434]	0.000	0.304	[0.255,0.360]	0.000
	M_CN_	0.430	[0.350,0.530]	0.000	0.498	[0.378,0.656]	0.000	0.355	[0.256,0.494]	0.000
	M_RN_	0.271	[0.215,0.338]	0.000	0.319	[0.230,0.423]	0.000	0.219	[0.154,0.315]	0.000
PC→AN→CSC	M_AN_	−0.221	[−0.254,−0.190]	0.000	−0.230	[−0.280,−0.187]	0.000	−0.207	[−0.257,−0.167]	0.000
	M_CN_	−0.207	[−0.256,−0.168]	0.000	−0.184	[−0.257,−0.128]	0.000	−0.229	[−0.300,−0.72]	0.000
	M_RN_	−0.157	[−0.197,−0.125]	0.000	−0.158	[−0.216,−0.116]	0.000	−0.158	[−0.218,−0.109]	0.000
AS→CSC	M_AN_	-	-	-	-	-	-	-	-	-
	M_CN_	−0.095	[−0.200,−0.003]	0.040	−0.125	[−0.287,0.006]	0.065	−0.069	[−0.218,0.051]	0.298
	M_RN_	0.082	[0.004,0.154]	0.037	0.066	[−0.045,0.173]	0.231	0.093	[−0.020,0.192]	0.110
PC→CSC	M_AN_	−0.077	[−0.126,−0.026]	0.005	−0.051	[−0.124,0.015]	0.128	−0.111	[−0.187,−0.033]	0.005
	M_CN_	−0.093	[−0.148,−0.034]	0.004	−0.103	[−0.176,−0.020]	0.019	−0.084	[−0.164,0.000]	0.051
	M_RN_	−0.154	[−0.200,−0.102]	0.000	−0.133	[−0.196,−0.068]	0.001	−0.174	[−0.249,−0.098]	0.000
Total Ind	M_AN_	0.125	[0.093,0.160]	0.000	0.148	[0.098,0.199]	0.000	0.098	[0.053,0.147]	0.000
	M_CN_	0.222	[0.146,0.309]	0.000	0.314	[0.200,0.445]	0.000	0.126	[0.033,0.244]	0.004
	M_RN_	0.114	[0.069,0.170]	0.000	0.161	[0.090,0.247]	0.000	0.062	[0.004,0.139]	0.035

## Data Availability

The data that support the outcomes of this study are accessible from the corresponding author. Data are available from the authors with the permission of Tianjin University.

## Supplementary Materials

The following supporting information can be downloaded at: https://www.mdpi.com/article/10.3390/bs14050415/s1, Table S1: Measurement invariance test for children’s self-concept scale; Table S2: Multi-group analysis fitness and model invariance test table; Table S3: Multi-group analysis across gender fitness and model invariance test table.

## References

[1] Li F., Cui Y., Li Y., Guo L., Ke X., Liu J., Luo X., Zheng Y., Leckman J.F. (2022). Prevalence of mental disorders in school children and adolescents in China: Diagnostic data from detailed clinical assessments of 17,524 individuals. J. Child. Psychol. Psychiatry..

[2] The 2023 Blue Book of China’s Mental Health Released. https://health.gmw.cn.

[3] Wang X., Wang X., Ma H.B. (1999). Manual of Mental Health Rating Scale.

[4] Trautwein U., Lüdtke O., Köller O., Baumert J. (2006). Self-esteem, academic self-concept, and achievement: How the learning environment moderates the dynamics of self-concept. J. Pers. Soc. Psychol..

[5] Su L., Wan G., Yang Z., Luo X., Li X., Liu M., Li Z., Huang C., Wang M. (1994). The Revising of Piers-Harris’s Self-concept Scale in Hunan Province. Chin. J. Clin. Psychol..

[6] Bronfenbrenner U., Ceci S.J. (1994). Nature-nurture reconceptualized in developmental perspective-a bioecological model. Psychol. Rev..

[7] Yin X. (2004). A Review of Research on the Relationship between Family Parenting Styles and Child Development. Stud. Presch. Educ..

[8] Deci E.L., Ryan R.M. (1985). The general causality orientations scale: Self-determination in personality. J. Res. Personal..

[9] Ryan R.M., Deci E.L. (2017). Self-Determination Theory: Basic Psychological Needs in Motivation, Development, and Wellness.

[10] Ryan R.M., Deci E.L. (2019). Brick by brick: The origins, development, and future of self-determination theory. Adv. Motiv. Sci..

[11] Ryan R.M., Deci E.L. (2006). Self-regulation and the problem of human autonomy: Does psychology need choice, self-determination, and will?. J. Personal..

[12] Ryan R.M., Deci E.L. (2000). Self-determination theory and the facilitation of intrinsic motivation, social development, and wellbeing. Am. Psychol..

[13] Vasquez A.C., Patall E.A., Fong C.J., Corrigan A.S., Pine L. (2016). Parent autonomy support, academic achievement, and psychosocial functioning: A meta-analysis of research. Educ. Psychol. Rev..

[14] Suh G.W., Fabricius W.V., Stevenson M.M., Parke R.D., Cookston J.T., Braver S.L., Saenz D.S. (2016). Effects of the interparental relationship on adolescents’ emotional security and adjustment: The important role of fathers. Dev. Psychol..

[15] Van der Kaap-Deeder J., Vansteenkiste M., Soenens B., Mabbe E. (2017). Children’s daily well-being: The role of mothers’, teachers’, and siblings’ autonomy support and psychological control. Dev. Psychol..

[16] Bean R.A., Northrup J.C. (2009). Parental psychological control, psychological autonomy, and acceptance as predictors of self-esteem in Latino adolescents. J. Fam. Issues.

[17] Brownell C.A.E., Kopp C.B.E. (2007). Autonomy, Compliance, and Internalization. Socioemotional Development in the Toddler Years: Transitions and Transformations.

[18] Joussemet M., Landry R., Koestner R. (2008). A self-determination theory perspective on parenting. Can. Psychol..

[19] Mageau G.A., Ranger F., Joussemet M., Koestner R., Moreau E., Forest J. (2015). Validation of the perceived parental autonomy support scale (P-PASS). Can. J. Behav. Sci..

[20] Deci E.L., Ryan R.M. (2000). The “what” and “why” of goal pursuits: Human needs and the self-determination of behavior. Psychol. Inq..

[21] Deci E.L., Ryan R.M. (2008). Self-determination theory: A macro theory of human motivation, development, and health. Can. Psychol..

[22] Deci E.L., Ryan R.M. (1987). The support of autonomy and the control of behavior. J. Pers. Soc. Psychol..

[23] Slemp G.R., Field J.G., Ryan R.M., Forner V.W., Van den Broeck A., Lewis K.J. (2024). Interpersonal supports for basic psychological needs and their relations with motivation, well-being, and performance: A meta-analysis. J Pers Soc Psychol..

[24] Luyckx K., Vansteenkiste M., Goossens L., Duriez B. (2009). Basic need satisfaction and identity formation: Bridging self-determination theory and process-oriented identity research. J. Couns. Psychol..

[25] Heissel A., Pietrek A., Flunger B., Fydrich T., Rapp M.A., Heinzel S., Vansteenkiste M. (2019). The validation of the German basic psychological need satisfaction and frustration scale in the context of mental health. Eur. J. Health Psychol..

[26] Xu D., Yu C., Dou K., Liang Z., Li Z., Nie Y. (2019). Parental autonomy support and adolescents’ future planning: The mediating role of basic psychological needs and personal growth initiative. Psychol. Dev. Educ..

[27] Neubauer A.B., Schmidt A., Kramer A.C., Schmiedek F. (2021). A little autonomy support goes a long way: Daily autonomy-supportive parenting, child well-being, parental need fulfillment, and change in child, family, and parent adjustment across the adaptation to the COVID-19 pandemic. Child. Dev..

[28] Kenny M.C., Adriana M. (2009). Children’s self-concept: A multicultural comparison. Prof. Sch. Couns..

[29] Gómez Pérez I.A., Gallardo-Montes C.D.P., Ballesta-Claver J., Ayllón Blanco M.F. (2023). Assessing Self-Concept in Children (Aged 5–7) with Functional Dyslalia. Children.

[30] Thomas M.B., Richard P.L. (1992). Self as Narrative: The Place of Life History in Studying the Life Span. The Self: Definitional and Methodological Issues.

[31] Marsh H.W. (1989). Age and sex effects in multiple dimensions of self-concept: Preadolescence to early adulthood. J. Educ. Psychol..

[32] Su L., Luo X., Zhang J., Xie G., Liu Y. (2002). Norms of the Piers-Harris Children’s Self-concept Scale of Chinese urban children. Chin. Ment. Health J..

[33] Chirkov V.I., Ryan R.M. (2001). Parent and Teacher Autonomy-Support in Russian and U.S. Adolescents. J. Cross Cult. Psychol..

[34] Wang Q., Pomerantz E.M., Chen H. (2007). The role of parents’ control in early adolescents’ psychological functioning: A longitudinal investigation in the United States and China. Child. Dev..

[35] Greenfield P.M., Keller H., Fuligni A., Maynard A. (2003). Cultural Pathways Through Universal Development. Annu. Rev. Psychol..

[36] Chao R.K. (1994). Beyond parental control and authoritarian parenting style: Understanding Chinese parenting through the cultural notion of training. Child. Dev..

[37] Zu Y. (2017). Senior High School Students’ Academic Delay of Gratification: Influencing Factors and Impact. Ph.D. Thesis.

[38] Gagné M. (2003). The role of autonomy support and autonomy orientation in prosocial behavior engagement. Motiv. Emotion..

[39] Liu J., Lin L., Lv Y., Wei C., Zhou Y., Chen X. (2013). Reliability and validity of the Chinese version of the Basic Psychological Needs Scale. Chin. Ment. Health J..

[40] Zhou H., Long L. (2004). Statistical Remedies for Common Method Biases. Adv. Psychol. Sci..

[41] Xiong H., Zhang J., Ye B., Zheng X., Sun P. (2012). Common Method Variance Effects and the Models of Statistical Approaches for Controlling It. Adv. Psychol. Sci..

[42] Cheung G.W., Rensvold R.B. (2002). Evaluating goodness-of-fit indexes for testing measurement invariance. Struct. Equ. Model..

[43] Wen Z., Ye B. (2014). Analyses of mediating effects: The development of methods and models. Adv. Psychol. Sci..

[44] Byrne B.M. (2010). Structural Equation Modeling with AMOS: Basic Concepts, Applications, and Programming (Multivariate Applications series).

[45] Rong T.S. (2010). AMOS and Research Methods.

[46] Wang J., Kaufman T., Mastrotheodoros S., Branje S. (2024). The Longitudinal Associations Between Parental Autonomy Support, Autonomy and Peer Resistance. J. Youth Adolesc..

[47] Lekes N., Gingras I., Philippe F.L., Koestner R., Fang J. (2010). Parental autonomy-support, intrinsic life goals, and well-being among adolescents in China and North America. J. Youth Adolesc..

[48] Grolnick W.S., Ryan R.M. (1989). Parent styles associated with children’s self-regulation and competence in school. J. Educ. Psychol..

[49] Ntoumanis N., Ng J., Prestwich A., Quested E., Hancox J., Thøgersen-Ntoumani C., Deci E., Ryan R., Lonsdale C., Williams G.C. (2021). A meta-analysis of self-determination theory-informed intervention studies in the health domain: Effects on motivation, health behavior, physical, and psychological health. Health Psychol. Rev..

[50] Froiland J.M., Worrell F.C. (2017). Parental autonomy support, community feeling and student expectations as contributors to later achievement among adolescents. Educ. Psychol..

[51] Froiland J.M. (2015). Parents’ weekly descriptions of autonomy supportive communication: Promoting children’s motivation to learn and positive emotions. J. Child. Fam. Stud..

[52] Piers E.V., Harris D.B. (1964). Age and other correlates of self-concept in children. J. Educ. Psychol..

[53] Demo D.H. (1992). The self-concept over time: Research issues and directions. Annu. Rev. Soc..

[54] Diseth A., Samdal O. (2014). Autonomy support and achievement goals as predictors of perceived school performance and life satisfaction in the transition between lower and upper secondary school. Soc. Psychol. Educ..

[55] Deng L., Gao S., Zhao H., Wang X., Fang X. (2020). The influencing mechanism of autonomy support, basic psychological needs satisfaction on internalizing and externalizing problems of first-year vocational high School students. Stud. Psych. Behav..

[56] Shnabel N., Ullrich J. (2016). Putting emotion regulation in context: The (missing) role of power relations, intergroup trust, and groups need for positive identities in reconciliation processes. Psychol. Inq..

[57] Ku J.H., Ahn D. (2015). The relationship among family environment, basic psychological needs, and school engagement of upper elementary school students in Korea. Korean J. Youth Stud..

[58] Simões F., Alarcão M. (2014). Promoting well-being in school-based mentoring through basic psychological needs support: Does it really count?. J. Happiness Stud..

[59] Costa S., Cuzzocrea F., Gugliandolo M.C., Larcan R. (2015). Associations between parental psychological control and autonomy support, and psychological outcomes in adolescents: The mediating role of need satisfaction and need frustration. Child. Indic. Res..

[60] Iyengar S.S., Lepper M.R. (1999). Rethinking the value of choice: A cultural perspective on intrinsic motivation. J. Pers. Soc. Psychol..

[61] Taylor I.M., Lonsdale C. (2010). Cultural Differences in the Relationships Among Autonomy Support, Psychological Need Satisfaction, Subjective Vitality, and Effort in British and Chinese Physical Education. J. Sport. Exerc. Psy..

[62] Smetana J.G. (1995). Conflict and coordination in adolescent-parent relationships. Child. Dev..

[63] Cohen S., Wills T.A. (1985). Stress, social support, and the buffering hypothesis. Psychol. Bull..

[64] Ahl R.E., Fausto-Sterling A., García-Coll C., Seifer R. (2013). Gender and discipline in 5–12-month-old infants: A longitudinal study. Infant. Behav. Dev..

[65] Bumpus M.F., Crouter A.C., McHale S.M. (2005). Parental autonomy granting during adolescence: Exploring gender differences in context. Dev. Psychol..

[66] Wang Q., Chan H.W., Lin L. (2012). Antecedents of Chinese parents’ autonomy support and psychological control: The interplay between parents’ self-development socialization goals and adolescents’ school performance. J. Youth Adolesc..

[67] Wood J.J., McLeod B.D., Sigman M., Hwang W.C., Chu B.C. (2003). Parenting and childhood anxiety: Theory, empirical findings, and future directions. J. Child. Psychol. Psyc..

[68] Zhai M., Cui W., Wu Q., Xu F. (2024). Prediction of Parental Psychological Control on Children’s Anxiety: The Buffering Effect of the Other Parent-child Closeness. Chin. J. Clin. Psychol..

[69] Damon W., Hart D. (1982). The development of self-understanding from infancy through adolescence. Child. Dev..

[70] Rosenberg M. (1986). Self-Concept from Middle Childhood through Adolescence. Psychological Perspectives on the Self.

