# Supplementary material for: The Relationship between Parental Autonomy Support and Children’s Self-Concept in China—The Role of Basic Psychological Needs
Source: Behav Sci (Basel). 2024 May 15;14(5):415. doi: 10.3390/bs14050415 (PMC11117511; doi:10.3390/bs14050415)
Supplement: Supplementary file 1 [file behavsci-14-00415-s001.zip › behavsci-2940517-supplementary.pdf]

Table S1. Measurement invariance test.

Group	Measurement Model	CMIN/DF	RMSEA	CFI	SRMR	Models	ΔCFI	ΔRMSEA
Gender	1 Unconstrained	6.939	0.044	0.993	0.017			
	2 Measurement weights	6.417	0.042	0.991	0.021	2 vs. 1	-0.002	-0.002
	3 Structural covariances	6.077	0.040	0.991	0.022	3 vs. 2	0.000	-0.002
	4 Measurement residuals	8.197	0.048	0.978	0.021	4 vs. 3	-0.013	0.008
Grade	1 Unconstrained	6.340	0.041	0.994	0.0163			
	2 Measurement weights	5.911	0.040	0.991	0.0228	2 vs. 1	-0.003	-0.001
	3 Structural covariances	5.861	0.040	0.991	0.0273	3 vs. 2	0.000	0.000
	4 Measurement residuals	10.350	0.055	0.971	0.0207	4 vs. 3	-0.02	0.015

Table S2. Multi-group analysis fitness and model invariance test table.

Model	CMIN/DF	RMSEA	NFI	CFI	GFI	ΔCMIN	ΔDF	*p*	Δ NFI	ΔIFI
M1	5.790	0.039	0.977	0.981	0.969	-	-	-	-	-
M2	5.585	0.038	0.976	0.980	0.967	29.418	9	0.001	0.001	0.001
M3	5.512	0.038	0.975	0.980	0.967	37.794	12	0.000	0.001	0.001
M4	5.465	0.038	0.975	0.980	0.967	37.974	13	0.000	0.001	0.001
M5	5.169	0.037	0.974	0.979	0.965	76.271	27	0.000	0.003	0.003

[1] M1 (Unconstrained Model), M2 (Measurement weights Model), M3 (Structural weights Model), M4 (Structural covariances Model), M5 (Measurement residuals Model).

Table S3. Multi-group analysis across gender fitness and model invariance test table.

	Model	CMIN/DF	RMSEA	NFI	CFI	GFI	ΔCMIN	ΔDF	*p*	Δ NFI	ΔIFI
	M1	5.467	0.038	0.968	0.973	0.961	-	-	-	-	-
	M2	5.309	0.037	0.966	0.973	0.960	31.549	11	0.001	0.001	0.001
M_{AN}	M3	5.224	0.037	0.966	0.972	0.960	37.149	15	0.001	0.001	0.001
	M4	5.184	0.037	0.966	0.972	0.960	45.234	18	0.000	0.002	0.002
	M5	5.060	0.036	0.964	0.971	0.957	107.890	35	0.000	0.004	0.004
	M1	9.913	0.054	0.943	0.948	0.930	-	-	-	-	-
	M2	9.515	0.052	0.942	0.948	0.929	37.731	11	0.000	0.001	0.001
M_{CN}	M3	9.308	0.052	0.941	0.947	0.929	47.278	16	0.000	0.002	0.002
	M4	9.204	0.051	0.941	0.947	0.928	55.650	19	0.000	0.002	0.002
	M5	8.768	0.050	0.939	0.945	0.926	123.295	36	0.000	0.004	0.004
	M1	8.526	0.049	0.950	0.956	0.940	-	-	-	-	-
	M2	8.245	0.048	0.949	0.955	0.939	43.385	11	0.000	0.002	0.002
M_{RN}	M3	8.070	0.048	0.949	0.955	0.939	52.485	16	0.000	0.002	0.002
	M4	7.985	0.047	0.948	0.954	0.939	60.697	19	0.000	0.002	0.002
	M5	7.621	0.046	0.946	0.953	0.936	122.349	36	0.000	0.004	0.004

[1] M1 (Unconstrained Model), M2 (Measurement weights Model), M3 (Structural weights Model), M4 (Structural covariances Model), M5 (Measurement residuals Model).
